# The role of maternal gut microbiota in prenatal depression and offspring neurodevelopment

**DOI:** 10.3389/fnut.2026.1958794

**Published:** 2026-10-07

**Authors:** Qiushuang Jia, Chengqian Diao, Shuai Liu, Huilin Yang, Xia Chen, Liemin Ruan, Zhongze Lou

**Affiliations:** 1 Department of Psychosomatic Medicine, The First Affiliated Hospital of Ningbo University, Ningbo, Zhejiang, China; 2 ZhiHe Innovation Hub, Ningbo Key Laboratory of Human Microbiome and Precision Medicine, Zhejiang-Australia Joint Laboratory on Human Microbiome Research, Central Laboratory of the First Affiliated Hospital of Ningbo University, Ningbo, Zhejiang, China; 3 Department of Obstetrics and Gynecology, The First Affiliated Hospital of Ningbo University, Ningbo, Zhejiang, China; 4 School of Brain Science and Brain Medicine, Zhejiang University, Hangzhou, Zhejiang, China

**Keywords:** gut-brain axes, maternal gut microbiota, maternal-fetal interaction, microbial metabolite, offspring neurodevelopment, prenatal depression, short-chain fatty acids

## Abstract

Prenatal depression is a prevalent complication during pregnancy that not only adversely affects maternal mental health and elevates the risk of postpartum depression but also has enduring implications for offspring development. Alterations in the host metabolome and gut microbiome have been linked to the onset and progression of depression; however, the biological mechanisms underlying the association between maternal mood disorders and fetal brain development remain inadequately understood. Recent studies have associated prenatal depression and psychological stress with alterations in the maternal gut microbiota, microbial metabolism, and immune signaling. However, most human evidence remains observational, and it remains unclear whether these microbial alterations precede depressive symptoms, arise as a consequence of depression or psychological stress, or act as intermediate factors within the maternal gut–brain axis. These findings offer novel strategies for the prevention and treatment of threats to maternal-fetal health. This article systematically reviews recent advancements in research concerning the “microbiota-gut-brain” axis in the context of maternal-fetal health. It investigates the roles and mechanisms of gut microbiota through preclinical and clinical studies, assesses the feasibility of potential therapeutic strategies, and suggests directions for future research. The aim is to provide a theoretical foundation for interventions targeting maternal-fetal health in cases of prenatal depression.

## 1 Introduction

Antenatal depression (AD), a prevalent complication during pregnancy, adversely affects maternal mental health and heightens the risk of postpartum depression, while also having long-term implications for offspring development. A large-scale individual participant data meta-analysis including 76,514 children from seven European birth cohorts confirmed that prenatal maternal depression is significantly associated with increased internalizing, externalizing, attention-deficit/hyperactivity disorder (ADHD), and autism spectrum disorder symptoms in offspring, with no significant sex differences observed. These associations are largely independent of pre-pregnancy depression and are partially mediated by postnatal depression, highlighting prenatal depression as a key developmental risk factor for children (1). Epidemiological research consistently reveals associations between prenatal depressive symptoms and increased risks of emotional regulation disorders, behavioral abnormalities, and mood disorders in offspring (2, 3). These findings are consistent with the broader developmental programming hypothesis, which posits that intrauterine exposure factors can have lasting effects on neurobehavioral outcomes. Globally, the prevalence of perinatal depression is estimated to be approximately 17.7%. In China, a large multicenter cross-sectional survey of 100,020 women in the third trimester across 27 public hospitals in 11 provinces, municipalities, and autonomous regions reported a prevalence of 25.8% for possible antenatal depression (EPDS >9) and 11.4% for probable antenatal depression (EPDS >12). Significant geographical variation was observed, with the North showing the highest prevalence (30.8 and 15.7%, respectively) and the East the lowest (24.5 and 10.6%, respectively) (4). Despite the substantial threat posed by AD to both maternal and infant health, effective treatment strategies are limited. This limitation is primarily due to the dynamic physiological changes occurring during pregnancy, which complicate the understanding of the underlying mechanisms of AD, as well as significant individual variability and widespread environmental factors. The current understanding of the precise biological mechanisms and pathological pathways of AD remains inadequate, presenting significant challenges for early screening, prevention, and treatment initiatives.

Prenatal depression has traditionally been linked to neuroendocrine dysregulation involving the hypothalamic–pituitary–adrenal (HPA) axis and maternal immune activation (5). These mechanisms may be part of a broader biological network through which maternal psychological stress affects maternal physiology and the intrauterine environment. The maternal gut microbiota has emerged as another potential factor involved in prenatal depression (6).

Studies in pregnant women have reported associations between prenatal stress or depressive symptoms and alterations in gut microbial composition (7, 8). The gut microbiome communicates with the host through immune, endocrine, neural, and metabolic pathways (9, 10). Microbiota-derived metabolites such as short-chain fatty acids (SCFAs) may also participate in gut–brain communication and the regulation of inflammatory and metabolic processes (11, 12). Maternal gut dysbiosis may therefore influence maternal physiology and the biological environment relevant to fetal development.

Studies in wild rodent models have shown that prenatal stress can induce depression-like states in pregnant females and is associated with anxiety- and depression-like behaviors, impaired cognitive function, and alterations in hippocampal neurogenesis and dendritic spine density in offspring (13). Prenatal stress has also been associated with alterations in maternal microbial communities that may influence offspring microbial colonization and behavioral outcomes (5).

Rather than being a passive bystander, the gut microbiome constitutes a metabolically active and immunologically engaged ecosystem that participates in bidirectional signaling with the brain through neural, immune, and endocrine pathways (9, 10). Maternal gut microbial communities undergo dynamic changes across gestation, with marked differences observed between early and late pregnancy (14). Psychological stress or depressive symptoms may further modify maternal microbial patterns during pregnancy (7, 8, 15, 16). Longitudinal human studies have begun to demonstrate associations between prenatal stress exposure and shifts in both maternal and infant microbiota profiles, supporting the concept of a shared maternal–infant microbial continuum (17). Experimental data further suggest that psychological stress during gestation can mediate the vertical transmission of altered microbial communities, with measurable consequences for offspring neurochemical and behavioral outcomes (18).

Notably, immune dysregulation—long considered central to prenatal depression—may interact with alterations in the maternal gut microbiota. Recent mechanistic studies suggest that stress-associated microbial changes may contribute to inflammatory imbalance and neurodevelopmental vulnerability in the offspring (19). These findings support potential interactions between maternal inflammation and microbiota-related changes, although the direction and causal nature of these relationships remain uncertain in humans.

Collectively, these findings advocate for a reconceptualization of prenatal depression, not merely as a psychological condition, but as a multifaceted ecological disturbance within the interconnected maternal-fetal biological system. Recent integrative models underscore the potential role of the maternal microbiome as a pivotal regulatory node that interlinks stress physiology, immune signaling, and neurodevelopmental programming (9). Investigating this interface may thus yield crucial insights into the intergenerational transmission of psychiatric vulnerability.

The maternal–infant microbial continuum describes the link between maternal microbiota and infant microbiome development across the perinatal period. Maternal microbiota may influence the fetus during pregnancy through microbial metabolites and immune signals, while microbial transmission occurs mainly during and after birth through delivery, breastfeeding, maternal contact, and the early-life environment. Delivery mode, antibiotic exposure, maternal dysbiosis, and feeding practices further shape infant microbial colonization during the first 1,000 days of life (17, 20). This continuum extends from prenatal microbial influences to postnatal microbial transmission and colonization rather than representing a single event of vertical transmission.

## 2 Clinical evidence linking prenatal depression and microbiota

Research in this domain is still in its nascent stages, yet emerging patterns are beginning to coalesce. Clinical studies suggest that the association between maternal psychological symptoms and gut microbiota may change during pregnancy. Longitudinal studies have linked maternal stress, anxiety, and depressive symptoms with changes in gut microbial composition across early, middle, and late pregnancy (7, 8). These differences across gestational stages highlight the importance of microbiome sampling time when comparing studies. Although specific taxa vary across different populations and analytical methodologies, consistent findings include reductions in microbial richness and alterations in bacteria associated with SCFAs production. Fecal microbiota transplantation studies have shown that microbiota from individuals with depression can induce depression-like behavioral and neurobiological changes in recipient rodents (21). This provides experimental evidence that depression-associated microbiota can affect behavioral phenotypes in animals, but causality in humans remains unclear.

Fecal microbiota transplantation from women with prenatal depression into germ-free mice induces depressive-like behaviors and hippocampal neuroinflammation, accompanied by decreased Ligilactobacillus, increased Akkermansia, elevated plasma LPS levels, and activation of NF-κB/TNF-α/IL-6 signaling (22). These findings provide experimental evidence that prenatal depression-associated microbial communities can transmit depression-related behavioral and neuroinflammatory phenotypes in a murine host. However, because these causal effects have been demonstrated in animal recipients rather than in humans, they should not be interpreted as direct evidence that gut dysbiosis causes prenatal depression in pregnant women.

Recent research further substantiates this relationship. For instance, women experiencing prenatal depression demonstrate significant alterations in gut microbial composition, characterizes by an increase in *Akkermansia* and a decrease in *Ligilactobacillus*, which are associated with inflammatory markers such as IL-6 (23). Furthermore, fecal microbiota from women with prenatal depression has been shown to induce depressive-like behaviors and neuroinflammatory changes in germ-free mice, providing experimental evidence that depression-associated microbial communities can influence behavioral and neuroinflammatory phenotypes in a murine host (22). Animal models receiving microbiota transplants from depressed individuals also exhibit behavioral changes, underscoring the role of gut microbiota in modulating neuropsychiatric symptoms (24).

The significance of these findings is further elucidated when considering neonatal outcomes. Maternal emotional symptoms have been correlated with variations in neonatal meconium microbiota (2, 25), suggesting that microbial patterns during gestation may influence or at least correlate with early colonization processes. Additionally, maternal depressive symptoms have been associated with reduced fecal immunoglobulin A levels in infants (26), indicating altered immune-microbiota interactions during early life.

Significantly, fecal microbiota obtained from mothers experiencing prenatal depression has been demonstrated to induce depressive-like behaviors and hippocampal neuroinflammation when transferred into germ-free mice (22). Although this does not conclusively establish that analogous mechanisms occur in humans, it enhances the biological plausibility of observed clinical associations.

A multicenter prospective cohort study of 2,053 pregnant women linked antenatal depressive symptoms to maternal gut microbiota alterations and impaired offspring neurodevelopment. Analysis of 504 maternal fecal samples showed that antenatal depressive symptoms were associated with reduced butyrate-producing bacteria and altered amino acid metabolism linked to poorer infant cognitive outcomes (27).

Most human studies are observational and cannot establish the timing or causal direction of the relationship between gut microbiota and depressive symptoms. Maternal diet, obesity, socioeconomic status, medication use, especially antidepressants, physical activity, pregnancy complications, and psychological stress may confound these associations. Gut microbial changes may occur before depressive symptoms, result from depression or psychological stress, or act as intermediate factors in the bidirectional gut–brain relationship. Cross-sectional studies cannot determine which change occurs first because depressive symptoms and microbial profiles are assessed at the same time. Longitudinal studies with repeated assessments and control of major confounding factors are needed to clarify these relationships (28, 29).

Current evidence shows associations between prenatal depressive symptoms and gut microbial changes in humans, while experimental studies support causal effects of depression-associated microbiota in animals. Direct causality in humans remains unclear and requires longitudinal studies. FMT also has limitations because it transfers whole microbial communities and other biological components, making specific effects difficult to identify. Germ-free animals cannot fully represent human pregnancy, which limits the translation of FMT findings to prenatal depression. To facilitate comparison across evidence levels, the available human and experimental studies are summarized separately in Tables 1, 2, with emphasis on study design, sample size, microbiome assessment methods, key findings, and major limitations.

**Table 1:** Summary of human studies on maternal depression, gut microbiota, and offspring neurodevelopment.

Model	Sample size and unit	Measurements	Verified interpretation	Limitations	References
Human observational study; prenatal depressive symptoms	29 women with prenatal depressive symptoms and 27 controls.	16S rRNA gene sequencing (V3–V4); circulating cytokines; EPDS.	No significant group differences in alpha or beta diversity. *Intestinibacter* was implicated in a statistical mediation analysis involving IL-17A and depressive symptoms.	Observational mediation does not establish a biological causal pathway; full methods UV.	(6)
Longitudinal human pregnancy pilot study	40 enrolled; 35 analyzed. Fecal-sample subsets across the five visits: 25, 20, 21, 18 and 10.	Rectal and vaginal swabs; 16S rRNA sequencing; maternal and cord-blood immune measurements.	Stress/depression–microbiota associations varied across pregnancy and postpartum; maternal CCL2 was associated with late-pregnancy psychological measures.	Small, changing sample sizes; rectal and vaginal findings must be distinguished.	(7)
Longitudinal human pregnancy cohort	98 enrolled; 87 analyzed; 225 stool samples (78 early, 77 middle and 70 late pregnancy).	Shotgun metagenomics; anxiety and depression assessments.	Reported microbiota associations did not remain significant after correction for multiple comparisons.	Exploratory observational evidence; avoid presenting uncorrected associations as robust taxonomic changes.	(8)
Human mother–child cohort	410 mother–child pairs: 152 with maternal depressive symptoms and 258 without; 287 children followed at 24 months.	Meconium 16S sequencing; ASQ-3 and SDQ; statistical mediation analyses.	Prenatal depressive symptoms were associated with neonatal microbiota and subsequent child outcomes; richness and *Lactobacillus* were evaluated as statistical mediators.	Abstract-verified; full adjustment strategy and sample availability by assay UV. Mediation is not experimental causality.	(2)
Longitudinal human mother–infant cohort	160 enrolled; 151 dyads with at least one stool sample included.	Shotgun metagenomics; maternal distress measures.	Maternal distress showed time-dependent associations with maternal/infant microbiome features, including infant microbiome volatility.	No direct child neurodevelopmental endpoint in this study; associations differ by sampling time.	(17)
Human pregnancy–neonatal observational study	100 mother–infant dyads.	16S sequencing; psychological measures; salivary cortisol.	Maternal depression was associated with lower neonatal alpha diversity; cortisol was associated with beta diversity.	Abstract-verified; no later child neurodevelopmental outcome established here.	(25)
Human observational study; prenatal depressive symptoms	86 women: 43 with depressive symptoms (EPDS ≥10) and 43 controls.	16S sequencing (V3–V4); targeted fecal SCFAs; plasma serotonin.	Fecal acetate, propionate and butyrate were higher in the depressive-symptom group; alpha and beta diversity did not differ significantly.	Fecal concentrations cannot be equated with systemic exposure or placental transfer; observational design.	(50)

**Table 2:** Summary of experimental studies on maternal stress/depression, gut microbiota, and offspring neurodevelopment.

Model	Sample size and unit	Measurements	Verified interpretation	Limitations	References
Pregnancy psychological stress in Wistar rats	12 PPS dams and 12 control dams; offspring sample size varied by endpoint (fecal metagenomics: *n* = 5/group; PFC inflammatory measurements: *n* = 8/group)	Shotgun metagenomics; untargeted fecal metabolomics; prefrontal neurochemical and inflammatory measurements	Prenatal psychological stress was associated with microbial and metabolic alterations in dams and offspring, together with depression-like behavior and neurochemical/inflammatory changes in offspring.	Offspring sample size varied by endpoint; shared or co-altered taxa and correlation analyses do not establish strain-resolved vertical transmission or direct causality.	(18)
Human-to-mouse FMT; non-pregnant female germ-free C57BL/6 recipients	Human donors: 6 prenatal-depression and 6 control donors, pooled by group; mouse recipients: 7/group (14 total)	FMT; cecal shotgun metagenomics; untargeted metabolomics; plasma inflammatory measurements; hippocampal immunofluorescence and qPCR	PD-donor FMT induced depression-like phenotypes, increased microbial richness, altered microbial composition and metabolism, elevated plasma LPS, and increased hippocampal microglial proliferation and inflammatory transcripts.	Recipients were non-pregnant and offspring were not studied. Increased TNF-α and IL-6 were demonstrated at the hippocampal mRNA level rather than as increased plasma concentrations; transcript abundance alone does not establish pathway activation.	(22)
Prenatal restraint-stress model in Sprague–Dawley rats	Pregnant dams: PS *n* = 9, CON *n* = 6; male offspring sample size varied by endpoint (microbiome: *n* = 6/group; FST: *n* = 8/group)	16S rRNA gene sequencing; untargeted fecal metabolomics; sucrose preference and forced-swim tests	Prenatal stress increased susceptibility to depression-like behavior in male offspring and was associated with altered gut microbial diversity and metabolic pathways, particularly glycerophospholipid and glycerolipid metabolism.	Only male offspring were studied; sample size varied by endpoint, and microbiome–metabolome associations do not establish a direct causal mechanism.	(30)
Prenatal restraint-stress model in Sprague–Dawley rats with gestational prolactin supplementation	Female offspring: *n* = 8/group; one offspring randomly selected per litter	Behavioral tests; PRL intervention; cAMP signaling and synaptic analyses; scRNA-seq; microglial analyses; 16S rRNA gene sequencing	Gestational PRL supplementation alleviated depression-like behavior, neuronal damage, neuroinflammation, synaptic dysfunction, and gut microbiota dysbiosis in prenatally stressed offspring and exerted neuroprotective effects through cAMP signaling.	Only female offspring were studied; the findings are based on an animal prenatal-stress model and cannot be directly extrapolated to human prenatal depression.	(32)
Human prenatal-depression donor FMT into germ-free female C57BL/6 mice	Human donors: 6 prenatal-depression and 6 control donors, pooled by group; mouse recipients: 8/group (16 total)	FMT; fecal metagenomic sequencing; LC–MS untargeted metabolomics; brain lipid metabolomics; behavioral tests	PD-donor FMT induced depression-like behaviors and was associated with altered gut microbial functions and central lipid metabolism, including increased glycerophospholipid and decreased sphingolipid metabolites.	Recipients were non-pregnant germ-free mice and offspring were not studied; pooled donor samples prevent assessment of donor-specific effects, and the findings do not establish causality in human prenatal depression.	(51)

## 3 Experimental evidence and mechanistic directionality

Rodent models offer the potential to advance beyond mere correlation. Prenatal stress paradigms reliably induce alterations in maternal microbial communities, which are accompanied by behavioral changes in offspring (18, 30, 31). Animal experiments have demonstrated that prenatal stress leads to a significant decrease in serum prolactin levels in mother rats, while exogenous prolactin supplementation during pregnancy can alleviate depressive-like behaviors in offspring, improve neuronal damage and synaptic plasticity in the medial prefrontal cortex, and exert neuroprotective effects by activating the cAMP/PKA/CREB signaling pathway, promoting microglial M2 polarization, and improving gut microbiota disorders (32). In certain models, stress-induced microbial modifications are transmitted vertically and are associated with changes in offspring neurotransmitter profiles18. These findings imply that maternal microbial ecology may play a role in early-life programming, rather than merely reflecting maternal physiology.

Prenatal probiotic intervention achieves effective vertical transmission from mother to infant, primarily through fecal/vaginal routes and early postnatal colonization. Such early probiotic establishment enables offspring to resist stress-induced pathogenic gut microbes, inhibits Th17-driven peripheral inflammation, normalizes gastrointestinal dysfunction and serotonin metabolism, and significantly alleviates autism-like behaviors including social deficits and hyperactivity. Mechanistically, prenatal probiotics suppress stress-induced neuroinflammation, reduce microglial overactivation, and prevent abnormal hypermyelination in the offspring brain. Furthermore, gut microbial metabolites such as tyrosol and xanthurenic acid directly exert anti-inflammatory effects, inhibit IL-17-induced myelination-related gene overexpression, and protect neural stem cells under stress, thereby contributing to improved neurodevelopmental outcomes (33).

Additional environmental perturbations further substantiate this perspective. Prenatal exposure to methamphetamine has been shown to alter offspring behavior through microbiota-regulated Wnt signaling pathways (34). Similarly, antibiotic exposure during gestation has been demonstrated to induce depressive-like phenotypes via a microbiota-STING pathway (35). Exposure to glyphosate and nanoplastics also disrupts gut-brain axis dynamics, resulting in emotional abnormalities in offspring (31, 36). Across these diverse models, a common theme emerges: disturbances to maternal microbial ecosystems during pregnancy are associated with long-term behavioral consequences.

Notably, maternal microbiota dysbiosis, even in the absence of overt psychological stress, can independently produce enduring behavioral alterations in offspring (24). These findings complicate efforts to isolate “depression” as a singular causal factor, instead highlighting microbial ecology as a shared mechanistic substrate across multiple gestational stressors.

Recent studies further underscore the role of maternal microbiota in shaping offspring neurodevelopment. For instance, one study demonstrated that maternal gut microbiota dysbiosis, induced by prenatal stress, leads to behavioral abnormalities in offspring, implicating alterations in gut-brain signaling pathways have been implicated as a fundamental mechanism (37). Furthermore, prenatal exposure to environmental factors such as antibiotics and pollutants has been demonstrated to modify the composition of the microbiota, leading to neurodevelopmental impairments, including behaviors resembling anxiety (38, 39). These findings underscore the critical importance of maintaining a balanced maternal microbiome for optimal fetal neurodevelopment. Additionally, recent evidence suggests that microbial metabolites, particularly SCFAs, play a pivotal role in modulating the fetal immune response and neurogenesis in the fetal brain, thereby presenting potential targets for alleviating stress-induced neurodevelopmental disorders (40).

## 4 Immune activation and neuroinflammatory signaling

Maternal gut microbial changes have been associated with immune dysregulation in prenatal depression (6). Animal studies suggest that prenatal stress and MIA can affect neuroinflammation and neurodevelopment, with microbiota-related immune pathways implicated in these effects (38). These experimental findings provide mechanistic evidence but cannot establish the same effects in human prenatal depression.

Among the proposed mechanism, immune activation has garnered the most sustained scholarly attention. Elevated levels of pro-inflammatory cytokines are frequently observed in conjunction with prenatal depressive symptoms, with maternal gut microbiota potentially mediating this inflammatory profile (6). Increased intestinal permeability and endotoxin exposure are plausible pathways through which microbial imbalance may exacerbate systemic inflammation. Longitudinal studies in humans further indicate that prenatal stress is associated with elevated IL-6 concentrations in offspring during childhood, suggesting that immune alterations may persist beyond gestation and contribute to subsequent vulnerabilities (41).

More specifically, animal studies have identified a microbiota–Th17–IL-17A pathway linking maternal immune activation to abnormal fetal neurodevelopment. In mouse models, maternal gut bacteria that promote Th17-cell differentiation can enhance IL-17A production following immune challenge, leading to abnormal cortical development and subsequent social and behavioral deficits in the offspring (38). Further mechanistic evidence suggests that IL-17A-dependent signaling can disrupt fetal microglial function and impair the development of cortical parvalbumin-positive (PV+) interneurons, thereby affecting inhibitory neural circuits and contributing to autism-like behavioral abnormalities (42). These findings provide a more specific mechanistic link between maternal gut dysbiosis, immune activation, and offspring neurodevelopment. However, this pathway has been demonstrated primarily in animal MIA models, and its relevance to human prenatal depression remains to be established.

MIA during pregnancy is widely acknowledged as a developmental risk factor for neuropsychiatric disorders (43). Inflammatory mediators can traverse or influence the placental environment, thereby shaping fetal immune programming and altering neurodevelopmental trajectories. Importantly, the placenta is not merely a passive barrier for maternal–fetal exchange but also functions as an active immunological and endocrine interface. Maternal microbiota-derived metabolites and inflammatory signals may modulate placental immune responses, vascular function, nutrient transport, and endocrine programs, thereby altering the intrauterine environment and fetal neurodevelopment (44). Thus, the placenta may serve as a key intermediary integrating maternal microbial signals before their effects are transmitted to the developing fetus. Experimental evidence shows that maternal gut microbiota can regulate immune responses at the maternal–fetal interface, including IFN-γ- and IL-17-related responses (45). These findings support the presence of a gut–placenta immune axis, although its specific role in prenatal depression remains unclear. Notably, inflammatory signaling does not function in isolation. Emerging research underscores mitochondrial bioenergetic systems as potential mediators that link maternal inflammation to disrupted brain development (46). Due to the role of mitochondria in regulating oxidative stress, calcium homeostasis, and apoptotic pathways, mitochondrial dysfunction induced by inflammation may exacerbate neural vulnerability during critical periods of synaptic organization.

In the developing brain, inflammatory signals modulate microglial maturation, synaptic pruning, and neuronal survival. Experimental evidence in dicates increased hippocampal neuroinflammation in offspring subjected to prenatal stress-related microbial alterations (24). Converging evidence from clinical and translational research further supports the notion that neuroinflammation server as a common pathway across multiple neurodevelopmental disorders associated with maternal immune activation (47).

## 5 Microbial metabolites as functional mediators

In addition to inflammation, microbial metabolites establish a direct biochemical connection between maternal gut ecology and fetal brain development. SCFAs, tryptophan derivatives, bile acids, and lipid mediators exert influence on neurodevelopment through both immune modulation and direct neural signaling (11, 12). Longitudinal neuroimaging studies have shown that maternal antenatal depression leads to sex-specific deviations in the normative development of the offspring’s thalamus and nucleus accumbens, which remain stable from early to late adulthood. Females show smaller volumes of these regions, while males show larger nucleus accumbens volumes, and these structural abnormalities are associated with increased depressive symptoms in adulthood (48). Multi-omics studies, which integrate 16S rRNA sequencing with metabolomics, reveal coordinated alterations in microbial composition and metabolic outputs in depressive states (49), highlighting functional metabolic shifts rather than merely taxonomic changes. Experimental evidence further supports a role for microbial metabolites in fetal neurodevelopment. In mice, the maternal microbiome regulates metabolites in maternal serum and the fetal brain, and supplementation with selected microbiota-dependent metabolites can restore impaired thalamocortical axon growth (37). These findings provide experimental evidence that microbiota-dependent metabolites can contribute to fetal brain development in mice.

Clinical evidence from a third-trimester pregnancy cohort further demonstrates that prenatal depression is closely linked to altered gut microbiota and functional metabolite profiles. Specifically, a higher abundance of Candidatus_Soleaferrea correlates with a lower risk of prenatal depression, while elevated propionic acid concentrations are associated with an increased risk. Moreover, plasma 5-hydroxytryptamine (5-HT) levels are significantly lower in depressed pregnant women, and 5-HT concentrations are positively correlated with the abundance of NK4A214_group and negatively correlated with unclassified_c_Clostridia, indicating that gut microbiota may modulate prenatal mood through regulating tryptophan metabolism and 5-HT biosynthesis (50).

Dysbiosis associated with maternal stress further disrupts lipid signaling pathways. Alterations in glycerophospholipid and sphingolipid metabolism have been implicated in modifications to membrane dynamics, receptor signaling, and synaptic function within mood-related brain regions (51, 52). Recent research by Pessa-Morikawa et al. (2022) demonstrated that microbial metabolites such as trimethylamine N-oxide (TMAO) and 5-aminovaleric acid betaine (5-AVAB) were significantly different between germ-free and specific pathogen-free mice, impacting fetal metabolic and neurodevelopmental pathways (53). These findings show that the maternal microbiota shapes metabolite profiles in the placenta and fetal brain, although the functional effects of individual metabolites remain unclear.

Importantly, current evidence supports a predominantly indirect mechanism whereby the maternal gut microbiome influences fetal neurodevelopment by regulating the maternal metabolic milieu rather than through direct microbial exposure of the fetus. Microbiota-regulated metabolites, including SCFAs and tryptophan derivatives, can reach the fetal compartment through the placenta and subsequently modulate neurodevelopmental processes (54–56). Thus, the placenta may serve as a critical interface linking maternal microbial metabolism to fetal brain development. FMT from women with antenatal depressive symptoms to germ-free dams caused gut dysbiosis and disrupted polyunsaturated fatty acid and amino acid metabolism in the fetal brain, resulting in cognitive deficits in the offspring. Maternal butyrate supplementation partially reversed these metabolic and neurodevelopmental changes and supported a role for microbiota-derived metabolites in the effects of antenatal depression on offspring neurodevelopment (27).

Furthermore, the study by Husso et al. (2023) elucidated the pivotal role of metabolites derived from the maternal microbiota in modulating gene expression within fetal tissues, particularly concerning immune and neural development in the fetal brain (57). These metabolites have the capacity to influence the modulation of the fetal immune system, thereby impacting long-term neurodevelopmental outcomes.

Tryptophan-derived metabolites, including IPA and kynurenine, are involved in immune regulation and neural signaling. Maternal tryptophan metabolism is associated with fetal developmental outcomes, but direct evidence linking specific microbiota-derived tryptophan metabolites to human fetal brain development remains limited (58). Alterations in microbial metabolites due to maternal dysbiosis may also disrupt neurotransmitter balance, which is essential for early brain development.

Additionally, Zhang et al. (2025) conducted a review on how maternal microbial dysbiosis influence the offspring’s risk of developing neurodevelopmental disorders, such as ASD, underscoring the significance of microbial metabolites in shaping fetal neurodevelopment through immune and metabolic pathways (59). This study underscores the impact of maternal gut microbiota dysbiosis on altering microbial metabolites, which may contribute to neuroinflammatory and neuroimmune disruptions linked to ASD.

In conclusion, Frerichs et al. (2024) proposed that microbial metabolites exert an influence on fetal neurodevelopment through the microbiota-gut-brain axis (MGBA), emphasizing the role of metabolites such as SCFAs, tryptophan derivatives, and bile acids in modulating brain development via immune activation and neural signaling pathways (60). These findings further underscore the critical role of maternal gut microbiota in shaping the neurodevelopmental trajectory of offspring.

Collectively, these insights support a convergent model wherein microbial metabolites and inflammatory signals collaboratively influence neuronal plasticity. Elucidating the temporal and causal relationships among these factors is crucial for understanding vulnerabilities within the prenatal gut-brain axis (Table 3).

**Table 3:** Summary of microbiota-related evidence in prenatal psychological exposures and offspring outcomes.

Model	Changes in gut microbiota	Inflammatory factors	Mechanism	References
Human pregnancy cohorts	Composition varies across studies; no consistent diversity signature; fecal SCFAs ↑ in one cohort	Cytokine associations vary; IL-17A-related association reported in one study	Maternal distress, microbial alterations, and child outcomes are associated in selected cohorts; causality in humans remains unproven.	(2, 6, 8, 50)
Prenatal stress rat models	Maternal and offspring microbial communities altered; effects vary by sex and experimental endpoint	Offspring cortical IL-1β ↑ and IL-6 ↓ reported in one model	Prenatal stress is accompanied by maternal/offspring microbial and metabolic alterations and offspring behavioral changes; direct microbial mediation remains to be established.	(18, 30)
Non-pregnant germ-free female C57BL/6 mice receiving human PD-donor FMT	*Ligilactobacillus* ↓; *Akkermansia* ↑; microbial richness ↑	Plasma LPS ↑; hippocampal TNF-α and IL-6 mRNA ↑	PD-donor FMT transfers selected behavioral, microbial, metabolic, and neuroinflammatory phenotypes to non-pregnant recipient mice; offspring were not studied.	(22, 51)
Germ-free maternal mice receiving human AND (antenatal depression)-donor FMT	Donor-associated microbial alterations; placental butyrate ↓	Fetal-brain TNF-α ↑; fetal-brain IL-1β ↑	Maternal FMT affects fetal-brain and offspring outcomes; gestational butyrate supplementation partially ameliorates selected abnormalities in this model.	(27)
Drug-exposed rodent models	Exposure-specific dysbiosis; Lachnospiraceae ↓ and myristic acid ↓ in the relevant model	Microglial M1 polarization reported in one model	Separate exposure models implicate microbiota-associated Wnt signaling or myristic acid–ARF1–STING regulation rather than directly modeling prenatal depression.	(34, 35)
Maternal immune activation mice	Th17-inducing maternal gut bacteria, including SFB	Maternal Th17–IL-17A response	Microbiota-dependent immune signaling contributes to offspring abnormalities in MIA models; applicability to prenatal depression remains uncertain.	(38)

## 6 Pharmacological and probiotic modulation

The introduction of pharmacological treatments adds an additional layer of complexity. Selective serotonin reuptake inhibitors (SSRIs) administered during pregnancy can alter gut microbial composition and metabolic output (61, 62). While effective management of maternal depression may mitigate fetal exposure to stress hormones, the downstream effects of SSRI-induced microbial changes remain uncertain.

Clinical metabolomic evidence from a large pregnancy cohort further demonstrates that prenatal maternal anxiety is strongly associated with impaired neonatal neurobehavioral performance, as reflected by lower Neonatal Behavioral Neurological Assessment (NBNA) scores. Umbilical cord blood metabolomics reveals significant reductions in L-carnitine, mevalonate acid, and 3-hydroxy-3-methylglutaric acid in infants of anxious mothers; these metabolites are key regulators of the mevalonate-cholesterol pathway, which is essential for fetal brain myelin formation, synaptic function, and neurodevelopment. The imbalanced mevalonate-cholesterol metabolism mediates the adverse impacts of prenatal anxiety on offspring neurodevelopment, highlighting cholesterol homeostasis as a critical metabolic target for protecting fetal brain development under maternal mental stress (63).

Current evidence from randomized controlled trials suggests that probiotic supplementation may yield small but significant improvements in depressive and anxiety symptoms during pregnancy and the early postpartum period, with strain-specific effects observed for *Lactobacillus rhamnosus* HN001, *Limosilactobacillus* reuteri PBS072, and *Bifidobacterium breve*. However, the overall evidence base remains limited and heterogeneous, with no available data supporting the use of prebiotics or synbiotics for maternal mental health. Variability in probiotic strains, dosages, intervention durations, and outcome measures contributes to inconsistent findings, and most studies focus on symptom reduction rather than the prevention of clinical mental disorders (64).

Probiotic interventions have generated cautious optimism, with early trials indicating that specific strains may alleviate anxiety or depressive symptoms during pregnancy (65), and Lactocaseibacillus rhamnosus HN001 has been demonstrated affect postpartum anxiety, monoamine levels, and gut microbial profiles (66). However, these findings are still in the preliminary stages. The interpretation is complicated by variability in strain selection, dosing, and participant characteristics.

Recent studies have underscored the potential advantages of maternal probiotic supplementation. For instance, Lu et al. (2020) demonstrated that supplementation with *Lactobacillus acidophilus* and *Bifidobacterium infantis* during pregnancy suppressed inflammation and supported neuronal and oligodendrocyte development in offspring, suggesting a promising strategy for enhancing neurodevelopment (67). Similarly, Halemani et al. (2023) conducted a systematic review, summarizing the mixed evidence regarding the effects of probiotics on maternal mental health and neonatal microbiota, while highting the importance of the gut-brain axis (68). This underscores the necessity for more targeted research to fully elucidate the impact of probiotics during pregnancy. Additionally, Binda et al. (2025) reported that probiotic supplementation during the third trimester and early lactation reduced the number of infection days in both mothers and infants, while promoting beneficial infant gut microbiota and potentially enhancing immune function (69).

An important but largely unexplored question is whether microbiome-targeted interventions could be used as adjuncts to pharmacotherapy in pregnant women who require antidepressant treatment. Because SSRIs can alter gut microbial composition and metabolic output (61, 70), probiotics may theoretically help maintain microbial homeostasis or mitigate microbiome-associated effects of pharmacological treatment. Notably, randomized clinical trials in non-pregnant patients with major depressive disorder have provided preliminary evidence that probiotics can be administered as adjuncts to ongoing antidepressant treatment, including fluoxetine, with potential additional improvement in depressive symptoms (71, 72). However, these findings cannot yet be extrapolated to pregnancy, and there is currently insufficient evidence to conclude that probiotics can reduce SSRI-associated maternal or fetal adverse effects, permit lower antidepressant doses, or delay or replace necessary pharmacotherapy during pregnancy (71). These possibilities require direct evaluation in prospective randomized trials comparing pharmacotherapy alone with combined pharmacotherapy and strain-specific probiotic interventions. Such studies should assess maternal psychiatric outcomes, gut microbial and metabolic profiles, antidepressant exposure and dosage, adverse events, pregnancy outcomes, and long-term offspring development. Until such evidence is available, probiotics should be considered investigational adjuncts rather than substitutes for indicated antidepressant treatment, with maternal disease control and maternal–fetal safety remaining the primary considerations.

Rather than viewing probiotics as universal remedies, future research should focus on identifying functional microbial signatures associated with resilience. Precision-based approaches, grounded in mechanistic insights, are likely to be more effective than broad-spectrum modulation.

## 7 Neurodevelopmental consequences

Long-term outcomes provide a sobering context As prenatal stress has been linked to persistent alterations in the development of the adolescent prefrontal cortex -a region critical for executive function and emotional regulation (3). A large population-based cohort study using distributed lag models showed that cumulative parental depression from the prenatal period to offspring adulthood increases the risk of depression and anxiety in offspring. Notably, only maternal prenatal depression is associated with offspring psychosis, while paternal prenatal depression has no such effect, emphasizing the unique importance of the prenatal period for maternal mental health (73). These alterations are particularly significant in the context of maternal stress, which has been shown to influence both the structure and function of brain regions responsible for cognition and emotional regulation. Georgousopoulou et al. (2024) further explored this issue, noting that prenatal stress can disrupt prefrontal cortex development and highlighting the differential effects based on sex, with males exhibiting greater vulnerability to stress-related brain changes (74).

Mounting evidence indicates that SCFAs—the key metabolites of gut microbiota—serve as critical regulators linking maternal gut dysbiosis to neurodevelopmental disorders such as ASD. Butyrate consistently exerts neuroprotective and anti-inflammatory effects by inhibiting histone deacetylase (HDAC) and suppressing NF-κB signaling, thereby reducing pro-inflammatory cytokines (IL-1β, IL-6, TNF-α) and promoting microglial polarization toward an anti-inflammatory phenotype. Acetate has different effects on inflammation depending on the immune environment. GPR43-mediated acetate signaling can reduce inflammation but may promote inflammatory responses under specific conditions (75, 76). Supplementation with a single SCFA may also disrupt the balance among acetate, propionate, and butyrate and cause unintended effects. In contrast, propionate predominantly promotes neuroinflammation, induces astrogliosis, activates the AKT pathway, and triggers social deficits, cognitive impairment, and autism-like behaviors in animal models. Distinct SCFA profiles and imbalanced SCFA-mediated neuroinflammation thus represent a core mechanistic pathway underlying ASD pathogenesis and adverse neurodevelopmental programming (77).

Endocrine factors, such as maternal prolactin, have been implicated in influencing depressive-like behaviors following prenatal stress (32), thereby, highlighting the interactions between hormonal and microbial systems. Prenatal stress-induced reductions in maternal prolactin levels are closely related to the occurrence of depressive-like behaviors in offspring, and prolactin supplementation can reverse these adverse outcomes by regulating the gut-brain axis (32). This interaction emphasizes how stress-induced hormonal changes can exacerbate the effects of maternal dysbiosis on the neurodevelopment of offspring. For example, maternal anxiety and stress during pregnancy have been associated with long-term behavioral issues in offspring, with the prefrontal cortex being particularly susceptible to these effects. Together, these observations suggest that prenatal stress may influence offspring neurodevelopment through interconnected hormonal, microbial, and neurobiological pathways, with potential long-term consequences for emotional and behavioral development.

Recent studies have furthered our understanding of these complex interactions. Suwaluk et al. (2022) demonstrated that prenatal stress not only alters immune responses but also disrupts neuroplasticity in the prefrontal cortex, leading to long-term cognitive and emotional challenges in offspring (3).

Collectively, these observations indicate that alterations in the maternal microbiota function within a comprehensive physiological network. These changes do not occur in isolation; rather, they intersect with immune, endocrine, and neurodevelopmental pathways, influencing trajectories of vulnerability. The complexity of these interactions necessitates the development of more sophisticated models to elucidate how maternal stress and microbiota alterations impact offspring brain development and emotional health throughout the lifespan (Figure 1).

**Figure 1:**
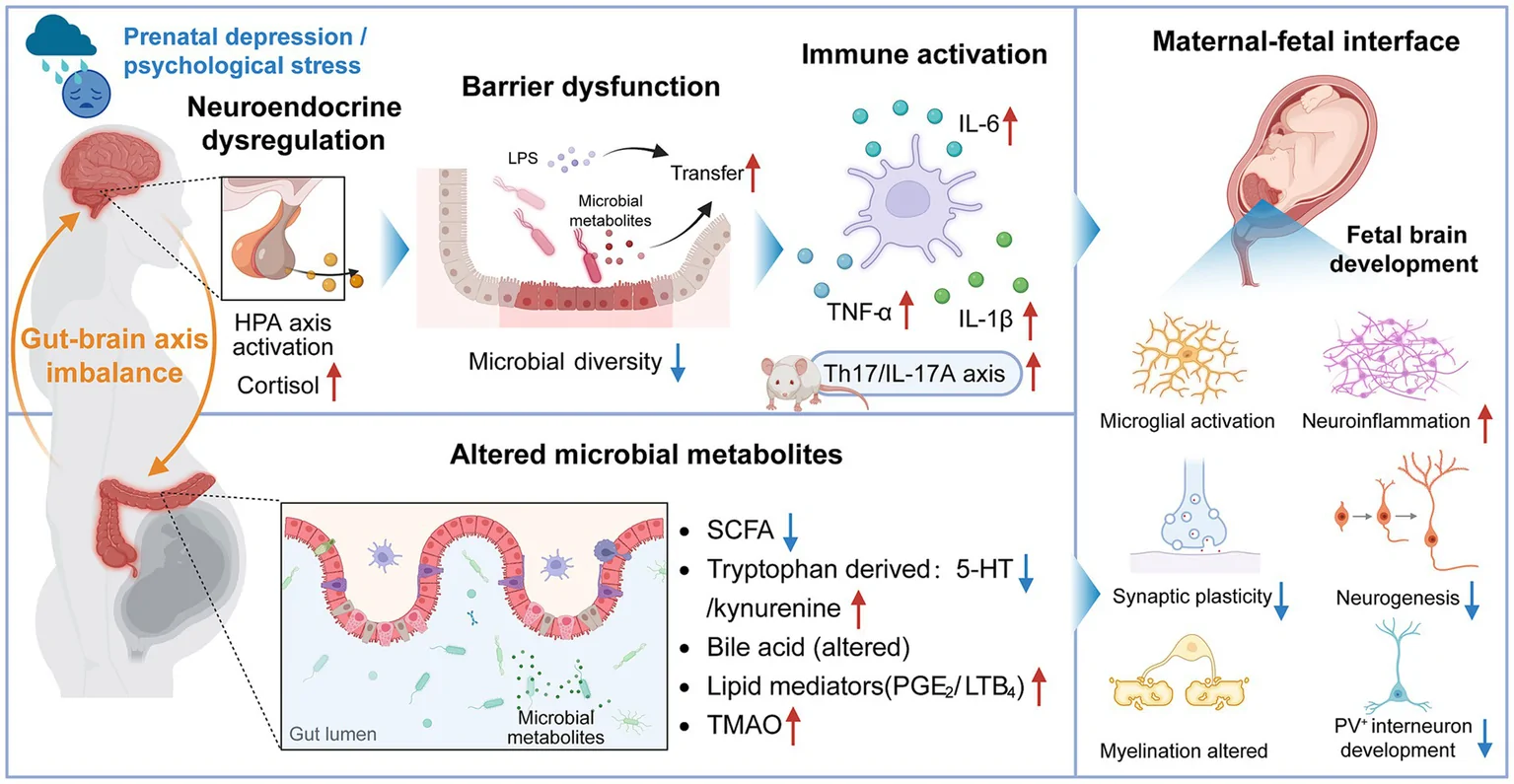
Proposed mechanisms linking maternal gut microbiota dysbiosis to offspring neurodevelopment in the context of prenatal depression. Maternal gut dysbiosis may influence offspring neurodevelopment through interconnected immune, metabolic, and neuroendocrine pathways at the maternal–fetal interface.

## 8 Conclusion

Current evidence supports an association between prenatal depression and alterations in the maternal gut microbiome (6, 10, 15), but the direction of this relationship remains uncertain. Maternal gut microbial alterations may contribute to depressive symptoms, may arise as a consequence of depression-related psychological and physiological changes, or may act as intermediate factors within a bidirectional gut–brain relationship. Human evidence remains predominantly observational, whereas stronger mechanistic evidence is largely derived from animal and FMT studies and should not be directly extrapolated to human prenatal depression. Microbiota-related immune and metabolic pathways may also contribute to the association between maternal psychological health and offspring neurodevelopment (6, 10, 15), although their causal roles in humans remain to be established.

A more nuanced comprehension of this interface may ultimately guide the development of preventive strategies to mitigate the burden of psychiatric disorders across generations. Future research should prioritize longitudinal cohort studies integrating metagenomic, metabolomic, and clinical phenotyping to clarify the temporal and causal relationships between maternal gut microbiota and prenatal depression. Rather than relying solely on taxonomic biomarkers, efforts should focus on defining functional microbial signatures associated with susceptibility or resilience to prenatal depression, including microbial metabolic pathways, community-level functions, and their interactions with host immune and neuroendocrine systems. Integration of microbiome profiling with targeted and untargeted metabolomics may further identify key bioactive mediators, such as butyrate and indole-3-propionic acid (IPA), and determine whether these metabolites represent mechanistic biomarkers or actionable therapeutic targets. Such functional characterization could provide a rational basis for developing strain-specific probiotics, dietary prebiotics, and personalized microbiome-targeted interventions.

At the same time, translation of microbiome-based interventions into pregnancy requires a stepwise framework that places maternal and fetal safety at the forefront. Postbiotics may offer a potentially more controllable approach by delivering defined microbial components or metabolites without administering viable microorganisms, whereas live biotherapeutic products require rigorous evaluation of strain identity, colonization dynamics, dosing, and reproductive safety. More invasive approaches, particularly fecal microbiota transplantation (FMT), should remain investigational in pregnant populations until their maternal-fetal safety is adequately established. Potential risks, including transmission of infectious agents, antimicrobial-resistance genes, unpredictable microbial engraftment, and unintended effects on fetal or neonatal microbial and immune development, require careful consideration. Future clinical translation should therefore incorporate predefined maternal and fetal safety endpoints, long-term follow-up of offspring, standardized microbiome and metabolome monitoring, and explicit ethical and regulatory oversight. Establishing this functional, mechanistic, and safety-oriented roadmap will be essential for translating microbiome research into precision strategies for preventing or treating prenatal depression.
